# Transcriptome Analysis Reveals the Dynamic and Rapid Transcriptional Reprogramming Involved in Heat Stress and Identification of Heat Response Genes in Rice

**DOI:** 10.3390/ijms241914802

**Published:** 2023-09-30

**Authors:** Yonggang He, Huimin Guan, Bo Li, Shuo Zhang, Yanhao Xu, Yan Yao, Xiaolong Yang, Zhongping Zha, Ying Guo, Chunhai Jiao, Haiya Cai

**Affiliations:** 1 Institute of Food Crops, Hubei Academy of Agricultural Sciences, Wuhan 430070, China; whuhyg@whu.edu.cn (Y.H.); guanhm99@163.com (H.G.); 201973043@yangtzeu.edu.cn (B.L.); zhangshuo@hbaas.com (S.Z.); xyh@hbaas.com (Y.X.); yy1045933811@163.com (Y.Y.); yxl0524@hbaas.com (X.Y.); zhongpingzha@163.com (Z.Z.); guokkkyyy@163.com (Y.G.); 2 Hubei Key Laboratory of Food Crop Germplasm and Genetic Improvement, Wuhan 430070, China; 3 Key Laboratory of Crop Molecular Breeding, Ministry of Agriculture and Rural Affairs, Wuhan 430070, China

**Keywords:** rice (*Oryza sativa* L.), heat tolerance, transcriptome, DEGs, transcription factor

## Abstract

High temperature is one of the most important environmental factors influencing rice growth, development, and yield. Therefore, it is important to understand how rice plants cope with high temperatures. Herein, the heat tolerances of T2 (Jinxibai) and T21 (Taizhongxianxuan2hao) were evaluated at 45 °C, and T21 was found to be sensitive to heat stress at the seedling stage. Analysis of the H_2_O_2_ and proline content revealed that the accumulation rate of H_2_O_2_ was higher in T21, whereas the accumulation rate of proline was higher in T2 after heat treatment. Meanwhile, transcriptome analysis revealed that several pathways participated in the heat response, including “protein processing in endoplasmic reticulum”, “plant hormone signal transduction”, and “carbon metabolism”. Additionally, our study also revealed that different pathways participate in heat stress responses upon prolonged stress. The pathway of “protein processing in endoplasmic reticulum” plays an important role in stress responses. We found that most genes involved in this pathway were upregulated and peaked at 0.5 or 1 h after heat treatment. Moreover, sixty transcription factors, including the members of the AP2/ERF, NAC, HSF, WRKY, and C2H2 families, were found to participate in the heat stress response. Many of them have also been reported to be involved in biotic or abiotic stresses. In addition, through PPI (protein–protein interactions) analysis, 22 genes were identified as key genes in the response to heat stress. This study improves our understanding of thermotolerance mechanisms in rice, and also lays a foundation for breeding thermotolerant cultivars via molecular breeding.

## 1. Introduction

Rice (*Oryza sativa* L.) is one of the most important cereal crops in the world and plays an increasingly significant role in global food security [1]; however, as the global temperature has been continuously increasing in recent years, extreme and high temperatures have adversely affected rice growth, yield, and grain quality [2]. It has been predicted that rice grain yields will decline by 10% for every 1 °C increase in daily maximum and minimum temperatures during the growing season, and continued warming could pose a serious threat to global production [3,4]. Rice plants are sensitive to high temperatures (>35 °C) at all growth stages, including the germination, vegetative, and reproductive stages [5]. At the seedling stage, high temperatures result in increased water loss, withered and yellow leaves, impaired seedlings and roots, and even seedling death [6]. At the reproductive stage, high temperatures lead to lower fertility and seed setting rates [7].

High temperatures influence the physiological and metabolic processes of rice. Under heat stress, the excessive accumulation of reactive oxygen species (ROS) in the chloroplast, mitochondria, and plasma membrane damages nucleic acids and proteins, adversely affects lipid peroxidation, and causes the release of harmful secondary metabolites [8,9]. To overcome this, plants have evolved various mechanisms with which to maintain ROS homeostasis, including antioxidant enzymes such as superoxide dismutase (SOD), ascorbate peroxidase (APX), peroxidase (POD), and catalase (CAT). It has been reported that improving the activity of CAT and SOD could enhance the thermotolerance of rice plants [10]. In addition, studies have proven that high temperatures could also influence photosynthesis, carbohydrate metabolism, and phytohormone balance in plants [6,11,12,13].

During its natural evolution and artificial domestication over time, rice has developed complex strategies with which to reduce damage and maintain normal growth under heat stress. Various signaling pathways, including Ca^2+^, NO, ROS, H_2_S, H_2_O_2_, kinase, G-protein, and plant hormone, have been reported to participate in heat sensing and signal transduction in rice [14,15,16,17]. Meanwhile, many HSPs (heat shock proteins) and transcription factors, including the members of the MYB, NAC, WRKY, HSF (heat shock transcription factor), and AP2/ERF families, are involved in transcriptional regulation in response to heat stimuli [18,19,20,21]. Among them, HSPs along with HSFs are the most important ones in heat response transcriptional regulatory networks, and these proteins are a response to the triggering of a transcriptional cascade to active downstream genes [22]. In addition, HSPs have an important role in protein folding/unfolding, protein transportation, cell signaling, and protection against stresses, including heat stress [23]. In rice, the overexpression of *OsHSP17.7* could increase the heat tolerance of the plants [24]. Although some signaling pathways involved in heat stress have been revealed in recent decades, there are still many details and new approaches yet to be uncovered.

To date, more than 80 rice heat response-related QTL (quantitative trait loci) have been identified through map-based cloning and reverse genetic technology. Among them, over 30 genes have been cloned and functionally characterized. *OsTT1*, identified in African rice (*Oryza glaberrima*), encodes an α2 subunit of the 26S proteasome involved in the degradation of ubiquitinated proteins [25]. The overexpression of *TT1^CG14^* could improve the heat tolerance of rice in the seedling, flowering, and filling stages. *TT2*, encoding a Gγ subunit, confers thermotolerance to rice plants at the vegetative and reproductive growth stages through the SCT1-dependent alteration of wax biosynthesis [26]. *TT3.1* and *TT3.2* interact to enhance the thermotolerance of rice seedlings and reduce the heat stress-related grain yield loss [27]. Furthermore, other genes, such as *OsMASD7*, *OsHTAS*, *OsANN1*, *HTH5*, *SCNA3*, *OsUBP21*, *OsFBN1*, *OsNTL3*, *OsMYB55*, *SNAC3*, *OsEDS1*, and *OsHSP90*, which encode proteases, heat shock proteins, channel proteins, and transcription factors, have been reported to be involved in rice heat tolerance [16,28,29,30,31,32]. These genes respond to heat stimuli through complex mechanisms, including hydrogen peroxide scavenging, unfolded protein renaturation, amino acid metabolism, Ca^2+^ signal transduction, and so on.

Transcriptomics provides highly accurate and cost-effective methods with which to reveal the transcriptome dynamics and molecular mechanisms of rice’s response to various stresses. According to the results, multiple genes and pathways involved in abiotic stimulus responses have been identified [33,34]. These studies help us understand how plants sense and respond to various stimuli. In the current study, to obtain more detail about the heat response pathways and genes, two rice germplasms were used as experimental materials, and their heat resistances were evaluated at 45 °C for 24 h. To elucidate the physiological changes in the two rice lines during heat treatment, the contents of H_2_O_2_ and proline, as well as the activities of some antioxidant enzymes, were investigated. Then, RNA-seq was used to reveal the dynamic transcriptome changes in rice seedlings by using samples at 0, 0.5, 1, 3, 8, and 12 h of heat treatment. Subsequently, the protein–protein interaction network (PPI) analysis was used to identify the hub genes in response to heat stimuli in the present study. The results of this study provide new insights into understanding the regulatory pathways involved in heat stress.

## 2. Results

### 2.1. Phenotype Characteristics of Two Rice Germplasms under Heat Stress

**Figure 1:**
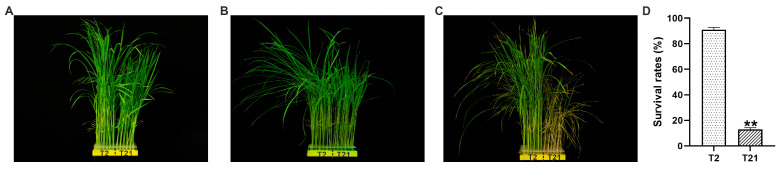
The morphologies of T2 and T21 seedlings before and after heat treatment. (**A**,**B**) Morphologies of T2 and T21 seedlings before (**A**) and after (**B**) heat treatment at 45 °C for 24 h. (**C**) Morphologies of T2 and T21 seedlings after they were recovered and kept under normal conditions for another 7 days. (**D**) The survival rates of T2 and T21 seedlings after heat treatment. Each experiment had three replicates, and more than 40 seedlings were tested in each replicate. Data represent the means ± SE. Significant differences were determined by using the Student’s *t*-test (** *p* < 0.01).

In this study, the heat tolerances of two rice germplasms, T2 (Jinxibai) and T21 (Taizhongxianxuan2hao), were evaluated at 45 °C for 24 h; they were subsequently recovered and kept under normal conditions for another 7 days (Figure 1A–C). We found that T2 exhibited high resistance to heat stress and the seedlings exhibited a survival rate of 90.93% after heat treatment (Figure 1C,D). In contrast, T21 showed a significantly lower survival rate of 12.92% (Figure 1C,D). These results revealed the different resistances of T2 and T21 to heat stress.

### 2.2. Effect of Heat Stress on Hydrogen Peroxide, Proline Content, and Antioxidant Enzyme Activity

**Figure 2:**
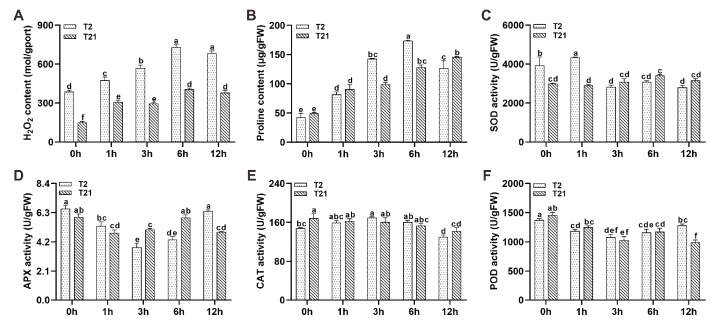
The physiological responses of T2 and T21 seedlings to the heat treatment. (**A**,**B**) The content of H_2_O_2_ (**A**) and proline (**B**) in the T2 and T21 seedlings after heat treatment. (**C**–**F**) The activity of SOD (**C**), APX (**D**), CAT (**E**), and POD (**F**) in the T2 and T21 seedlings after heat treatment. Values are means ± SE. The different lowercase letters indicate statistically significant differences according to a one-way ANOVA (*p* < 0.05).

In our study, we found that the content of H_2_O_2_ increased dramatically during heat treatment (Figure 2A). After heat treatment for 1, 3, 6, and 12 h, the content of H_2_O_2_ in T2 seedlings increased by 23.40%, 47.27%, 89.27%, and 76.96%, respectively; however, in the heat-sensitive germplasm, T21, its content was increased by 104.11%, 96.76%, 170.65%, and 151.95%, respectively. Proline is one of the most important osmoregulators in the cytoplasm of plants exposed to various abiotic stresses. We found that its content was dramatically induced by heat stress (Figure 2B). In T2 seedlings, the content of proline increased by 92.24%, 235.92%, 308.71%, and 198.35% after heat treatment for 1, 3, 6, and 12 h, respectively. The content of the T21 seedlings only increased by 83.51%, 100.68%, 160.07%, and 195.84% at each of abovementioned time points.

The activity of SOD increased by 10.39% after heat treatment for 1 h and then decreased in the T2 seedlings (Figure 2C); however, in the T21 seedlings, its activity did not significantly change (Figure 2C). The activity of APX was significantly decreased in the first 3 h and then rebounded from 6 to 12 h in the T2 seedlings (Figure 2D). As for the T21 seedlings, their activity decreased in the first 1 h and increased from 3 to 6 h, which was followed by a subsequent decrease in activity (Figure 2D). The activity of CAT increased slowly during the first 3 h and then decreased gradually in the T2 seedlings (Figure 2E); however, in the T21 seedlings, CAT activity remained unchanged during the first 3 h (Figure 2E). As for the activity of POD, we found that it decreased gradually from 0 to 3 h, followed by a slow increase in the T2 seedlings (Figure 2F); however, in the T21 seedlings, its activity decreased significantly from 6 to 12 h after heat treatment.

### 2.3. RNA-Seq Analysis and Identification of DEGs in Response to Heat Stress

To better understand the expression changes in the T2 and T21 seedlings under heat stress, we performed transcriptome profiling analysis on the seedlings subjected to heat treatment at 45 °C in a time course experiment (0, 0.5, 1, 3, 8, and 12 h). A total of 253.58 Gb of clean data was obtained from 36 libraries, each with a Q30 base percentage of 93.37% or higher (Table S1). The results of RNA sequence mapping showed that 90.15%–94.86% of clean reads could be successfully mapped onto the rice reference genome (MSU7.0). Additionally, 84.72%–92.66% of clean reads were uniquely mapped. Furthermore, the Pearson’s correlation coefficients among the three biological replicates in each sample varied from 0.8911 to 0.9697, indicating that the RNA-Seq data were highly reproducible. To further validate the reliability of the RNA-seq data, 10 genes were randomly selected for performing quantitative RT-qPCR in the current study. As shown in Figure S1, RT-qPCR detected a similar expression tendency to that of the RNA-seq data. These results demonstrated that the RNA-seq data used in the current study were highly reliable.

**Figure 3:**
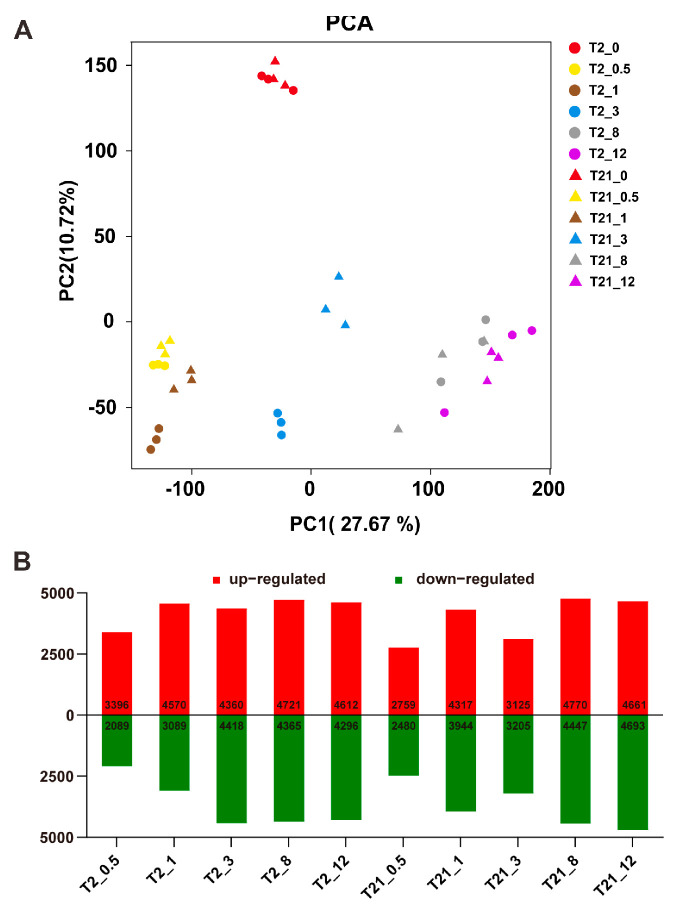
Overview of the transcriptome data and differentially expressed genes (DEGs) in the T2 and T21 seedling responses to heat stress. (**A**) Principal component analysis (PCA) of the expressed genes. (**B**) The number of upregulated and downregulated genes in the T2 and T21 seedlings after heat treatment for 0.5, 1, 3, 8, and 12 h.

As shown in Figure 3A, PCA (principal component analysis) manifested a tremendous difference between samples at 0 h and other time points (0.5, 1, 3, 8, and 12 h), indicating that heat stress induced large transcription level perturbations in rice seedlings. Then, based on the criteria of fold change ≥ 2 and a false discovery rate (FDR) < 0.01, the DESeq2 package was used to identify differentially expressed genes (DEGs) between the heat treatment and control in T2 and T21 at each time point. A large number of DEGs were identified in the two rice lines (Figure 3B). The number of upregulated genes was greater than that of the downregulated genes in the T2 seedlings after heat treatment for 0.5 and 1 h. Taken together, these data indicated that the heat treatment induced dramatic and dynamic transcriptional changes in rice.

### 2.4. The Basal Expression of Genes in T2 Differs from That in T21

**Figure 4:**
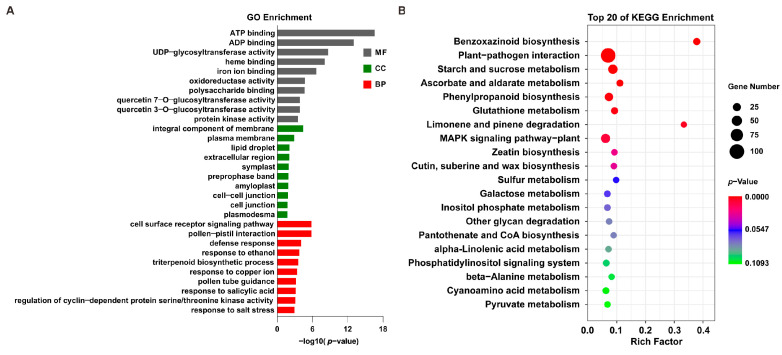
GO and KEGG analyses of the DEGs between T2 and T21 before heat treatment. (**A**) GO enrichment analysis of the 1995 DEGs between T2 and T21 after heat treatment for 0 h. (**B**) KEGG pathway enrichment analysis of the DEGs between T2 and T21 after heat treatment for 0 h.

To identify which genes were differentially expressed in the two rice lines at the basal level, we identified the DEGs between T2 and T21 before the heat treatment. Compared with T21, we found that 1995 genes were differentially expressed in T2 (1023 upregulated and 932 downregulated) (Figure S2). GO analysis showed that these DEGs related to biological processes were enriched in terms of “defense response”, “triterpenoid biosynthetic process”, “response to salicylic acid”, “response to salt stress”, “response to oxidative stress”, “response to heat”, and so on (Figure 4A). KEGG analysis indicated that the DEGs were enriched in “benzoxazinoid biosynthesis”, “plant–pathogen interaction”, “phenylpropanoid biosynthesis”, “MAPK signaling pathway–plant”, “glutathione metabolism”, and so on (Figure 4B). These results indicated that the biotic and/or abiotic stress response-related genes were differentially expressed in T2 and T21, which might be part of the reason for the differences in the heat sensitivities of the two germplasms.

### 2.5. Functional Annotation of DEGs in Response to Heat Stress 

**Figure 5:**
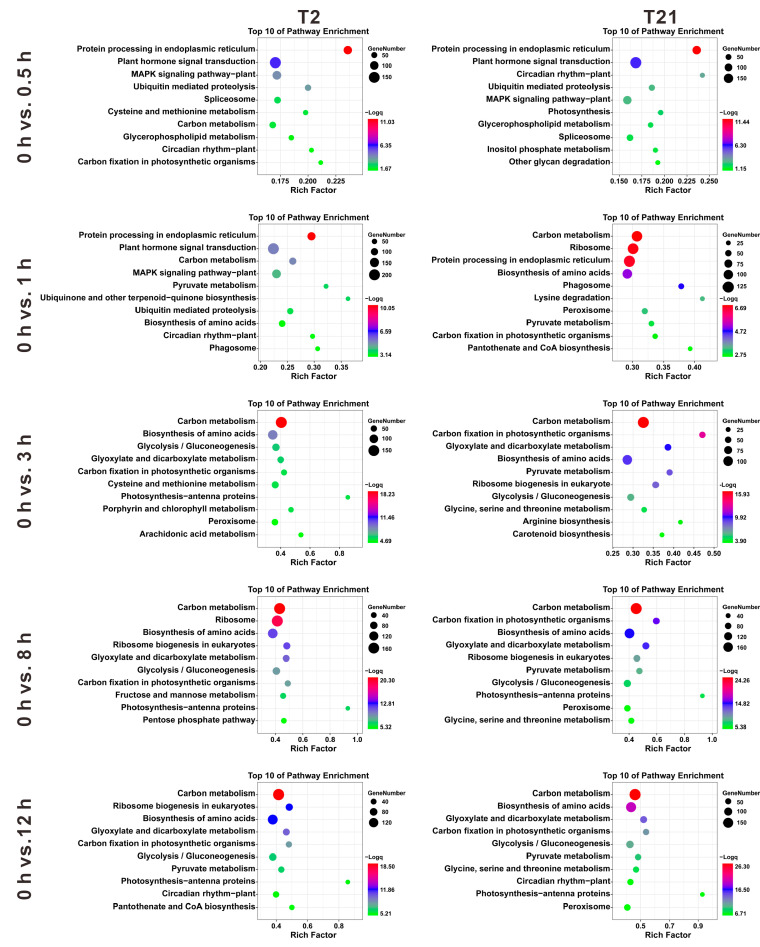
KEGG pathway enrichment analysis of the DEGs identified in T2 and T21 at different stress time points (0.5, 1, 3, 8, and 12 h).

KEGG pathway analysis was performed to reveal the thermal response mechanisms of T2 and T21. We found that the DEGs were significantly enriched (q-value < 0.05) in 18, 39, 52, 50, and 61 pathways in T2 at 0.5, 1, 3, 8, and 12 h, whereas 9, 43, 42, 49, and 58 significantly enriched pathways were found in T21, respectively. At the early stage (0.5 h), the DEGs in T2 and T21 were mainly involved in the KEGG pathways of “protein processing in endoplasmic reticulum”, “plant hormone signal transduction”, “MAPK signaling pathway–plant”, etc. (Figure 5). Moreover, the DEGs in T2 were also involved in “cysteine and methionine metabolism”, “carbon metabolism”, “carbon fixation in photosynthetic organisms”, and “plant–pathogen interaction” (Figure 5). After heat treatment for 1 h, the DEGs mainly participated in “carbon metabolism”, “MAPK signaling pathway–plant”, “pyruvate metabolism”, “carbon fixation in photosynthetic organisms”, “ubiquitin mediated proteolysis”, etc., in both T2 and T21 (Figure 5). 

After heat treatment for 3 h, KEGG analysis showed that the DEGs in T2 and T21 were involved in “biosynthesis of amino acids”, “glycolysis/gluconeogenesis”, “glyoxylate and dicarboxylate metabolism”, “photosynthesis-antenna proteins”, “porphyrin and chlorophyll metabolism”, etc. (Figure 5). Meanwhile, we found that similar pathways were activated after heat treatment for 8 and 12 h. These KEGG pathways include “glyoxylate and dicarboxylate metabolism”, “pyruvate metabolism”, “glycolysis/gluconeogenesis”, “serine and threonine metabolism”, “pentose phosphate pathway”, etc. (Figure 5). Taken together, these data indicated that the DEGs identified in T2 and T21 were enriched in similar pathways at each time point, and various pathways were activated as the duration of thermal stress increased.

### 2.6. Function Annotation of Common DEGs in T2 and T21

**Figure 6:**
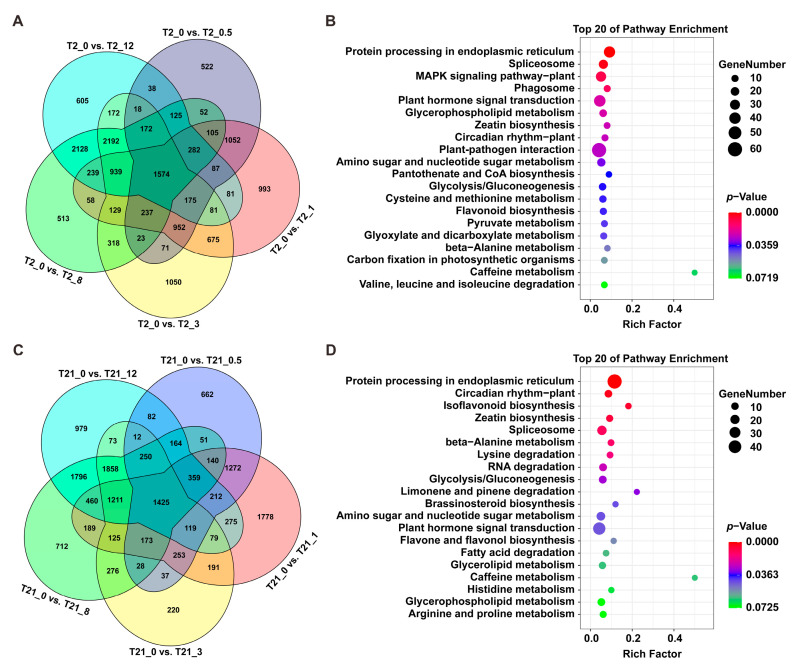
Functional analysis of the common DEGs in T2 and T21. (**A**) Overlap analysis of the DEGs identified in T2 after heat treatment for 0.5, 1, 3, 8, and 12 h. (**B**) KEGG enrichment analysis of the common DEGs in T2. (**C**) Overlap analysis of DEGs identified in T21 after heat treatment for 0.5, 1, 3, 8, and 12 h. (D) KEGG enrichment analysis of the common DEGs in T21.

Through a Venn diagram analysis, we identified 1574 and 1425 DEGs that were involved in the responses to heat stimuli at all five time points in T2 and T21, respectively (Figure 6A,C). Then, KEGG pathway analyses were performed to determine the pathway assignment of these DEGs. Remarkably, the 1574 common DEGs in T2 were enriched in some abiotic stress response-related pathways, including “protein processing in endoplasmic reticulum”, “MAPK signal pathway–plant”, “plant hormone signal transduction”, and “plant–pathogen interaction” (Figure 6B). The same KEGG enrichment analysis revealed that the 1425 DEGs in T21 were enriched in “protein processing in endoplasmic reticulum”, “spliceosome”, “plant hormone signal transduction”, and “limonene and pinene degradation” (Figure 6D). These results indicate that the genes of “protein processing in endoplasmic reticulum” and “plant hormone signal transduction” are involved in the response to heat stimuli during heat stress. We hypothesized that these genes might comprise a major part of the engine for transcriptional reprogramming during heat treatment.

### 2.7. The Pathway of Protein Processing in Endoplasmic Reticulum in Response to Heat Stress

**Figure 7:**
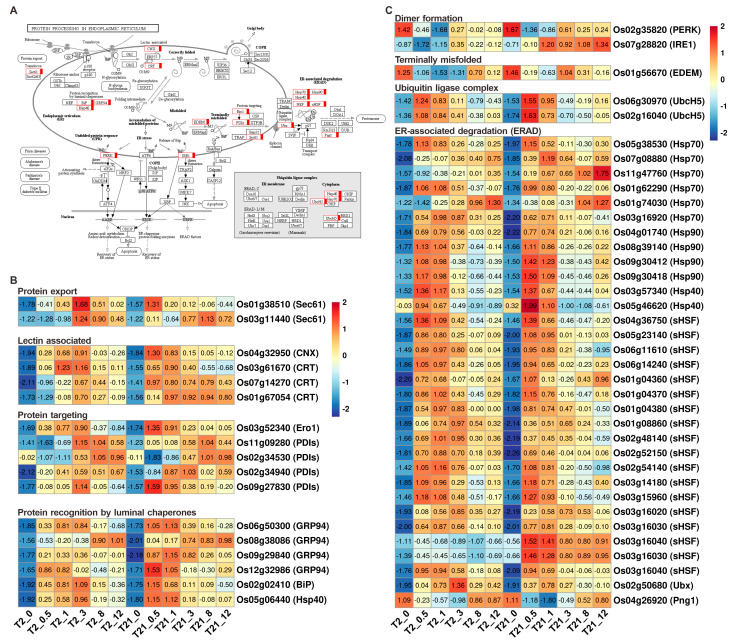
Protein processing in endoplasmic reticulum pathway and heatmap of the related genes. (**A**) The map of the protein processing in endoplasmic reticulum pathway. The red box represents the detected DEG-associated proteins. (**B**) The expression patterns of the DEGs involved in protein folding. (**C**) The expression patterns of the DEGs involved in protein degradation.

In total, we found that 54 genes participate in the pathway of “protein processing in endoplasmic reticulum”. These DEGs were mainly involved in protein translocation, protein folding and modification, and protein degradation (Figure 7A). After heat treatment, most of them were up-regulated immediately and peaked at 0.5 or 1 h (Figure 7B,C). There were 15 genes involved in the misfolded protein repair (*CNX*, *CRT*, *Ero1*, *PDIs*, *BiP*, *GRP94*, and *Hsp4*0) (Figure 7B). After heat treatment, all of these genes were up-regulated, except for *Os02g34530/OsPDIL5-3*. Meanwhile, we found that the expression levels of three calreticulin (CRT) genes (*Os03g61670*, *Os07g14270,* and *Os01g7054*) were significantly higher in T21 seedlings after heat treatment for 0.5 h, which is the same as those of *Os02g02410*/*OsBiP1* (Figure 7B). Besides this, the expression levels of some HSP members were also higher in T21 at the early heating stage, such as *Os06g50300*, *Os08g38086*, *Os09g29840*, *Os12g32986*, and *Os05g06440*, which are cofactors that regulate the binding of BiP to proteins or release BiP from the protein complex.

Furthermore, we found 32 genes involved in ER-associated degradation (ERAD) that are responsible for eliminating misfolded proteins (Figure 7A,C). These genes were upregulated across all stress time points in the two rice lines, except for *Os04g26920* (Png1). Meanwhile, we found 16 sHSPs (small heat shock proteins) and 12 HSPs involved in ERAD. These genes had similar expression patterns during heat treatment in T2 and T21 (Figure 7C). In addition, according to the heatmap, we found that the expression levels of *Os11g47760*, *Os03g16020*, and *Os03g16030* were significantly higher in T21 than those in T2.

### 2.8. Identification of Core Transcription Factors in Response to Heat Stimulus

**Figure 8:**
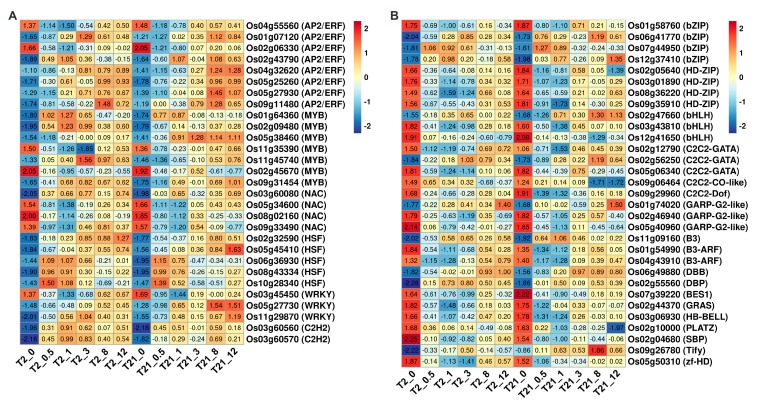
Heatmap displaying the expression profiles of the transcription factors. (**A**) The expression patterns of genes encoding the AP2/ERF, MYB, NAC, HSF, WRKY, and C2H2 family TFs. (**B**) The expression patterns of the other 31 TFs in T2 and T21.

From 1574 common DEGs (Figure 6A), a total of 125 DEGs encoding transcription factor (TF) family proteins were identified in T2 seedlings under continuous heat stress. These TFs were classified into 30 TF families, including AP2/ERF, WRKY, MYB, etc. The top six TFs families containing the greatest number of TFs were the AP2/ERF, MYB, NAC, bHLH, HSF, and bZIP families, including 17, 12, 10, 9, 8, and 7 genes, respectively. In T21, a total of 95 genes encoding TFs were identified from 1425 common DEGs, and most of them belonged to the AP2/ERF, MYB, HSF, HD-ZIP, bZIP, bHLH, and WRKY families. To identify the core TFs in response to heat stress, we compared the gene lists of the TFs described above. Sixty common genes were identified in T2 and T21, and about half of them were upregulated after heat treatment (Figure 8). Interestingly, after heating for 0.5 h, we found that the expression levels of most TFs were higher in T2 than those of T21 (Figure 8).

A total of eight AP2/ERF TFs were identified. After heat treatment, these genes were upregulated, except for *Os04g55560*/*SHAT1* (AP2/ERF-AP2) and *Os02g06630* (AP2/ERF-ERF) (Figure 8A). There were seven genes encoding MYB family members: five were upregulated and two were downregulated after heat treatment (Figure 8A); however, in the four *NAC* genes, only *Os03g60080*/*SNAC1*/*OsNAC9* was upregulated after heat treatment (Figure 8A). As for the five *HSFs* and three *WRKYs*, only *Os03g45450/OsWRKY60* was downregulated in response to heat stress. Furthermore, two C2H2 members (*Os03g60560* and *Os03g60570*) were upregulated immediately after heat treatment (Figure 8A). 

Other TFs, including the members of the bZIP, HD-ZIP, bHLH, and C2C2-GATA families, were also identified in the current study (Figure 8B). There were four genes encoding bZIP TFs: three genes were upregulated and one gene was downregulated (Figure 8B). The HD-ZIP TFs affect plant responses to abiotic stress and phytohormone signaling. We found that all four of the HD-ZIP TFs were downregulated in T2 and T21 after heat treatment (Figure 8B). The TFs in the bHLH and C2C2-GATA families have an important role in plant growth, and each of the three members was identified. These genes were downregulated in response to heat stress, except for *Os02g47660* (bHLH) and *Os02g56250* (C2C2-GATA) (Figure 8B). In addition, the TFs from the C2C2-CO-like, C2C2-Dof, GARP-G2-like, B3, and SBP families were also identified in the current study (Figure 8B).

### 2.9 Prediction of Protein–Protein Interactions (PPIs) to Identify the Hub Genes in Response to Heat Treatment

**Figure 9:**
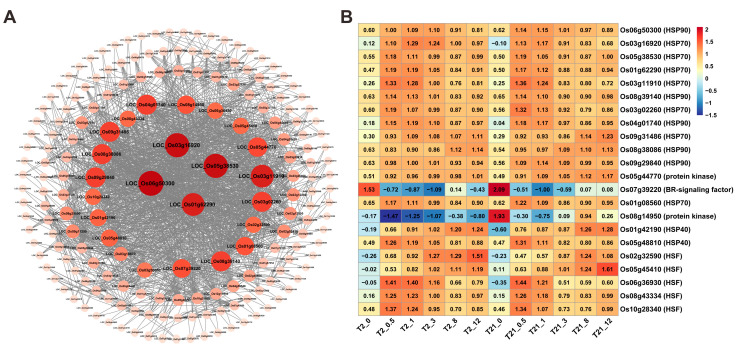
PPI analysis of the 797 common DEGs between T2 and T21. (**A**) Protein–protein interaction networks of the 797 common DEGs between T2 and T21 were constructed via the STRING database. (**B**) The expression patterns of the 22 hub genes in T2 and T21 under heat stress.

To identify which genes may play key roles in response to heat stress, we further overlapped the common genes (1574 DEGs in T21 and 1425 genes in T2, Figure 6A,C) and found that a total of 797 genes were involved in heat responses in both T2 and T21. Then, to identify which genes are located in core positions, the potential interactions between these 797 genes were investigated using the STRING database. In the PPI network, 22 hub genes with degrees from 26 to 51 were identified (Figure 9A). The expression patterns of these genes are depicted in the heat map (Figure 9B). In these hub genes, fourteen *HSPs* were upregulated quickly in T2 and T21 after heat treatment, which is the same as that of the five *HSFs.* In addition, two genes, *Os07g39220*/*OsBZR1* (BR signaling factor) and *Os08g14950* (receptor-like protein kinase 2 precursor), were also identified among the core genes, and were downregulated in response to heat stimuli. These hub genes may play a pivotal role in response to heat stress and downstream gene regulation.

## 3. Discussion

High temperature is a major abiotic stress that significantly affects rice plants’ growth, yield, and grain quality. In recent years, the frequency of extremely high-temperature climates has caused a great impact on rice growth [5,6]. Therefore, it is important to understand how rice plants withstand high temperatures. In the current study, the heat tolerance of two rice lines was evaluated (Figure 1). Thereafter, their physiological and biochemical characteristics were detected and RNA-Seq analyses were performed in a time course experiment to reveal their responding mechanisms.

Under heat stress, intracellular ROS levels are dramatically increased [35,36]. Despite the fact that the ROS could act as a signal to help plants cope with environmental stresses, overaccumulated ROS would lead to protein denaturation, damage to the membrane, and the impairment of antioxidant enzyme activity [6,8,37]. In this study, we also found that the content of H_2_O_2_ increased quickly after heat treatment, but the accumulation rate was significantly higher in T21. Meanwhile, heat stress induces the accumulation of proline in many plants [38], and plants with elevated proline levels exhibit enhanced tolerance to abiotic stresses [39]. In this study, we found that its content was increased significantly after heat stress and was higher in the heat-tolerant rice line T2. Therefore, the different contents and accumulation rates of H_2_O_2_ and proline may be part of the reason for the different heat sensitivities of T2 and T21. In addition, heat stress impairs the activities of antioxidant enzymes, especially SOD, CAT, and POD [36,40]. Similarly, we found that the activities of APX and POD were impaired, whereas CAT activity was relatively stable during heat treatment.

Heat stresses could induce several alterations at the gene expression level in plants [33,41]. In this study, we found that both T2 and T21 rapidly regulated gene expression in response to heat stimuli, but T2 activated more pathways than T21 after heat treatment for 0.5 h. It has been reported that the genes related to plant hormone signaling, calcium signaling, and protein repair can be rapidly activated in response to abiotic stress [42,43]. The same results were found in our study: “protein processing in endoplasmic reticulum”, “plant hormone signal transduction”, “MAPK signaling pathway – plant”, etc., were activated significantly during the early heating stage. These results are also similar to those of other studies: the pathway “MAPK signaling pathway–plant” has been shown to be activated after heat treatment for 10 min [41] and “protein processing in endoplasmic reticulum” plays an important role in maize seedlings to cope with heat stress [44]. Meanwhile, as the stress was prolonged, we also found that other pathways were activated, including “biosynthesis of amino acids”, “glyoxylate and dicarboxylate metabolism”, “pyrimidine metabolism”, and so on. These results revealed the complex mechanisms in rice plants responsible for defense against or adaptation to heat stress.

Heat stress disrupts the ER, especially by causing the accumulation of misfolded proteins, which activate the unfolded protein response [45]. In previous studies, the pathway of protein processing in the ER has been proven to play an important role in response to abiotic stress [44,46]. After heat treatment, the genes involved in this pathway, such as BiP, luminal chaperon (GRP94), protein folding machinery (CRT, CNX, PDIs, UGGT, and HSPs), protein-homeostasis-related sHSPs, and ERAD (Derlin, Ubx, UbcH5, HRD1, and SAR1), were induced in plants [41,44]. Similar results were obtained in the current study (Figure 5 and Figure 6). In plants, HSPs and sHSPs are known as molecular chaperones responsible for the events of protein folding, protein assembly, and degradation during heat stress [17,47]. We found that many members of HSP90, HSP70, HSP40, and sHSPs were dramatically upregulated after heat stress (Figure 7). Additionally, these HSPs have been reported to participate in protein folding, recognition, and degradation [17,48]. Altogether, these findings suggested that the genes involved in protein processing in the ER play an important role in rice’s ability to cope with heat stress.

TFs have been proven to participate in the regulation of heat stress responses in plants [49]. Under stress, TFs display a rapid response to environmental stimuli; afterward, these TFs drive changes in the expression of downstream genes to overcome or adapt to a given stress [50]. In this study, sixty TFs were identified, and these genes were up- or downregulated in response to heat treatment (Figure 8). Among them, most of the DEGs encoding AP2/ERF, MYB, HSFs, C2H2, and bZIP TFs were upregulated, whereas the opposite trends were found in NAC, HD-ZIP, bHLH, GARP-G2-like, and B3-ARF TFs (Figure 8). The AP2/ERF TF family contains four major subfamilies in plants, including AP2, RAV, ERF, and DREB. Many AP2/ERF genes have been reported to be involved in responses to a variety of environmental stimuli in plants [51,52]. Here, we identified eight DEGs encoding AP2, ERF, and SREB subfamily TFs, and four genes (*Os01g07120*/*OsDREB2A*, *Os02g43790*/*AP59*, *Os04g32620*/*OsERF10,* and *Os05g27930/OsDREB2B*) have been reported to be involved in the drought tolerance of rice [53,54,55,56]. Meanwhile, *Os09g11480*/*SUB1C* was reported to participate in the submergence response [57]. Moreover, the MYB, WRKY, bZIP, and C2H2 TFs control diverse biological processes, such as differentiation, development, and abiotic stress responses [58,59,60,61]. Likewise, we identified a total of 16 genes belonging to these families, and most of them were upregulated following heat treatment. Among these genes, *Os11g35390/OsMYB4P* is an R2R3-type MYB transcriptional activator that regulates the utilization of Pi in rice [62]; however, it was significantly downregulated after heat treatment (Figure 8A). This result may imply that heat stress could influence nutrient homeostasis in plants. As for the NAC TFs identified in this study, the overexpression of *Os03g60080*/*SNAC1* could enhance the drought tolerance of rice [63]. Similarly, the two C2H2 members (*Os03g60560* and *Os03g60570*) have been reported to be associated with salt and drought tolerance in rice [64,65]. Altogether, these observations suggested that TFs, such as AP2/ERF, MYB, WRKY, bZIP, and C2H2 members, play an important role in heat responses.

Notably, HSPs and HSFs are important components of plants’ responses to heat stress and are responsible for triggering transcriptional cascades and activating downstream genes such as ROS scavenging enzymes, metabolic enzymes, and HSPs [5]. According to the PPI network constructed by the 797 common DEGs, 19 genes encoding HSPs and HSFs were identified. Consistent with previous studies [66,67], we found that these genes were upregulated after heat treatment (Figure 9). In addition, previous studies have shown that *Os06g50300*/*OsHSP90* plays an important role in the innate immunity of rice [68,69]. The overexpression of *Os04g01740*/*OsHSP1* could improve the thermotolerance of *Arabidopsis* [70]. *Os09g31486* has been identified as a possible contributor to drought tolerance [71]. *Os08g43334*/*OsHsfB2b* negatively regulates the drought and salt tolerances of rice [72]. *Os05g45410*/*OsSPL7*/*OsHsf4d* plays a critical role in the balance of reactive oxygen species and stress responses in rice [73]. Therefore, the HSPs and HSFs identified in the current study may play central roles in plant responses to heat stress, and the genes identified from the PPI analysis may be good candidate genes for breeding heat-tolerant cultivars.

## 4. Materials and Methods

### 4.1. Plant Materials and Heat Tolerance Evaluation

The seeds of two indica rice cultivars with different tolerance levels to heat stress, Jinxibai (T2, heat-tolerant) and Taizhongxianxuan2hao (T21, heat-sensitive), were obtained from a large collection of rice accessions preserved at Hubei Province Crops Germplasm Resources Medium-Term Genebank, Wuhan, China. The sterilized seeds were soaked in water at 28 °C for 36 h, and subsequently germinated in Petri dishes containing wet filter papers. Then, the germinated seeds were planted in 96-well plates with one seed in each well. A total of 48 germinated seeds for each rice line were planted in the same plates. Then, the plates were transferred to float on a Yoshida nutrient solution. The solution was refreshed every 3 days, and the pH was adjusted to 5.5 daily. The culture conditions were as follows: 14 h/28 °C day and 10 h/25 °C night cycle. After 15 days, the seedings were subjected to heat stress treatment at 45 °C (RH = 75%) for 24 h in an artificial climate chamber (BOANTE, Hubei, China) and were then returned to normal conditions for recovery. The survival rates were calculated after a seven-day recovery period from the heat stress treatment. Each experiment had three replicates, and more than 40 seedlings were tested in each replicate.

### 4.2. Assay of H_2_O_2_ and Proline Level, and Enzyme Activity

After heat treatment, the leaf samples were harvested at 0, 1, 3, 6, and 12 h, immediately put in liquid nitrogen, and subsequently stored at −80 °C. The samples were homogenized in an ice-cold 0.1 M phosphate-buffered saline (PBS, pH 7.4) and then centrifuged at 8000 rpm for 10 min at 4 °C. The supernatants were used for biochemical analysis. The contents of H_2_O_2_ and proline, as well as the activities of SOD, APX, CAT, and POD, were investigated with commercial kits (Lot No.: A064-1-1, A107-1-1, A001-3, A123-1-1, A007-1-1, and A084-3-1) from the Nanjing Jiancheng Bioengineering Institute (Nanjing, China) according to the manufacturer’s protocols. All assays were performed with three biological and three technical replications.

### 4.3. RNA Extraction and RT-qPCR

After heat treatment for 0, 0.5, 1, 3, 8, and 12 h, the leaf samples were harvested and stored at −80 °C. Total RNA was extracted with TRIzol reagent (Invitrogen, Carlsbad, CA, USA) and then the qualified RNAs were used for transcriptome sequencing and RT-qPCR. First-strand cDNA was synthesized from 2 μg of RNA in a 20 μL reaction system with ABScript III RT Master Mix (ABclonal, Wuhan, China) according to the manufacturer’s instructions. Then, RT-qPCR was performed with a CFX96^TM^ real-time PCR Detection System (Bio-Rad, Hercules, CA, USA). The primers used in the qPCR are listed in Table S2. PCR thermal cycling conditions were 95 °C for 5 min, followed by 40 cycles of 95 °C for 10 s and 60 °C for 30 sec. All assays were performed with three biological and three technical replications. The OsActin gene expression served as an internal control to normalize the gene expression of the target genes.

### 4.4. RNA Sequencing and Data Analysis

A total of 36 cDNA libraries were constructed using the NEBNext Ultra RNA library Prep Kit for Illumina (NEB, Ipswich, MA, USA) following the manufacturer’s instructions. Paired-end 150 bp RNA-Seq was performed using an Illumina HiSeq 2500 platform (Illumina Inc, San Diego, CA, USA) by the Biomarker Technologies Corporation (Beijing, China). Thereafter, the generated reads were processed via Fastp software to remove low-quality reads, and the clean reads were aligned to the reference sequences of MSU v7.0 [74] using HISAT2. FPKM (fragments per kilobase of exon per million mapped fragments) was used to quantify the level of gene expression. Pearson’s correlation coefficient was used to evaluate the correlations of the FPKM of all transcripts among three replications for each sample. DEGs were identified via DESeq2 software [75], with the criteria of fold change ≥ 2 and a false discovery rate (FDR) < 1. The GO annotation and KEGG pathway analysis of the DEGs were performed using BMKCloud tools (www.biocloud.net, accessed on 26 March 2023), a free online platform for data analysis. In addition, the STRING database (https://string-db.org/, accessed on 3 April 2023) was used to analyze the protein–protein interaction (PPI) network, and Cytoscape v3.7.1 software was used to construct the predicted network graph.

## Data Availability

The raw data files of transcriptomic analysis have been uploaded to the NCBI Sequence Read Archive (SRA) database (Bioproject ID: PRJNA1014968).

## Supplementary Materials

The following supporting information can be downloaded at: https://www.mdpi.com/article/10.3390/ijms241914802/s1.

## References

[1] Wing R.A., Purugganan M.D., Zhang Q. (2018). The rice genome revolution: From an ancient grain to Green Super Rice. Nat. Rev. Genet..

[2] Krishnan P., Ramakrishnan B., Reddy K.R., Reddy V.R., Sparks D.L. (2011). Chapter three—High-temperature effects on rice growth, yield, and grain quality. Advances in Agronomy.

[3] Welch J.R., Vincent J.R., Auffhammer M., Moya P.F., Dobermann A., Dawe D. (2010). Rice yields in tropical/subtropical Asia exhibit large but opposing sensitivities to minimum and maximum temperatures. PNAS.

[4] Van Oort P.A.J., Zwart S.J. (2018). Impacts of climate change on rice production in Africa and causes of simulated yield changes. Glob. Chang. Biol..

[5] Li J.Y., Yang C., Xu J., Lu H.P., Liu J.X. (2023). The hot science in rice research: How rice plants cope with heat stress. Plant Cell Environ..

[6] Xu Y., Chu C., Yao S. (2021). The impact of high-temperature stress on rice: Challenges and solutions. Crop J..

[7] Jiang N., Yu P., Fu W., Li G., Feng B., Chen T., Li H., Tao L., Fu G. (2020). Acid invertase confers heat tolerance in rice plants by maintaining energy homoeostasis of spikelets. Plant Cell Environ..

[8] Waszczak C., Carmody M., Kangasjarvi J. (2018). Reactive oxygen species in plant signaling. Annu. Rev. Plant Biol..

[9] Lukan T., Coll A. (2022). Intertwined roles of reactive oxygen species and salicylic acid signaling are crucial for the plant response to biotic stress. Int. J. Mol. Sci..

[10] Qiao B., Zhang Q., Liu D., Wang H., Yin J., Wang R., He M., Cui M., Shang Z., Wang D. (2015). A calcium-binding protein, rice annexin OsANN1, enhances heat stress tolerance by modulating the production of H_2_O_2_. J. Exp. Bot..

[11] Huang G., Yang Y., Zhu L., Peng S., Li Y. (2021). Temperature responses of photosynthesis and stomatal conductance in rice and wheat plants. Agric. For. Meteorol..

[12] Mathur S., Agrawal D., Jajoo A. (2014). Photosynthesis: Response to high temperature stress. J. Photochem. Photobiol. B Biol..

[13] Wu C., Cui K., Wang W., Li Q., Fahad S., Hu Q., Huang J., Nie L., Peng S. (2016). Heat-induced phytohormone changes are associated with disrupted early reproductive development and reduced yield in rice. Sci. Rep..

[14] Finka A., Cuendet A.F., Maathuis F.J., Saidi Y., Goloubinoff P. (2012). Plasma membrane cyclic nucleotide gated calcium channels control land plant thermal sensing and acquired thermotolerance. Plant Cell.

[15] Xuan Y., Zhou S., Wang L., Cheng Y., Zhao L. (2010). Nitric oxide functions as a signal and acts upstream of *AtCaM3* in thermotolerance in *Arabidopsis* seedlings. Plant Physiol..

[16] Kan Y., Lin H.-X. (2021). Molecular regulation and genetic control of rice thermal response. Crop J..

[17] Saini N., Nikalje G.C., Zargar S.M., Suprasanna P. (2022). Molecular insights into sensing, regulation and improving of heat tolerance in plants. Plant Cell Rep..

[18] El-kereamy A., Bi Y.-M., Ranathunge K., Beatty P.H., Good A.G., Rothstein S.J. (2012). The rice R2R3-MYB transcription factor *OsMYB55* is involved in the tolerance to high temperature and modulates amino acid metabolism. PLoS ONE.

[19] Fang Y., Liao K., Du H., Xu Y., Song H., Li X., Xiong L. (2015). A stress-responsive NAC transcription factor *SNAC3* confers heat and drought tolerance through modulation of reactive oxygen species in rice. J. Exp. Bot..

[20] Wu X., Shiroto Y., Kishitani S., Ito Y., Toriyama K. (2009). Enhanced heat and drought tolerance in transgenic rice seedlings overexpressing *OsWRKY11* under the control of *HSP101* promoter. Plant Cell Rep..

[21] Ambavaram M.M.R., Basu S., Krishnan A., Ramegowda V., Batlang U., Rahman L., Baisakh N., Pereira A. (2014). Coordinated regulation of photosynthesis in rice increases yield and tolerance to environmental stress. Nat. Commun..

[22] Nadeem M., Li J., Wang M., Shah L., Lu S., Wang X., Ma C. (2018). Unraveling field crops sensitivity to heat stress: Mechanisms, approaches, and future prospects. Agronomy.

[23] Wang F., Zang X., Kabir M.R., Liu K., Liu Z., Ni Z., Yao Y., Hu Z., Sun Q., Peng H. (2014). A wheat lipid transfer protein 3 could enhance the basal thermotolerance and oxidative stress resistance of *Arabidopsis*. Gene.

[24] Murakami T., Matsuba S., Funatsuki H., Kawaguchi K., Saruyama H., Tanida M., Sato Y. (2004). Over-expression of a small heat shock protein, *sHSP17.7*, confers both heat tolerance and UV-B resistance to rice plants. Mol. Breed..

[25] Li X.M., Chao D.Y., Wu Y., Huang X., Chen K., Cui L.G., Su L., Ye W.W., Chen H., Chen H.C. (2015). Natural alleles of a proteasome α2 subunit gene contribute to thermotolerance and adaptation of African rice. Nat. Genet..

[26] Kan Y., Mu X., Zhang H., Gao J., Shan J., Ye W., Lin H. (2021). *TT2* controls rice thermotolerance through SCT1-dependent alteration of wax biosynthesis. Nat. Plants.

[27] Zhang H., Zhou J.F., Kan Y., Shan J.X., Ye W.W., Dong N.Q., Guo T., Xiang Y.H., Yang Y.B., Li Y.C. (2022). A genetic module at one locus in rice protects chloroplasts to enhance thermotolerance. Science.

[28] Zhang H., Xu H., Feng M., Zhu Y. (2018). Suppression of *OsMADS7* in rice endosperm stabilizes amylose content under high temperature stress. Plant Biotechnol. J..

[29] Cao Z., Tang H., Cai Y., Zeng B., Zhao J., Tang X., Lu M., Wang H., Zhu X., Wu X. (2022). Natural variation of *HTH5* from wild rice, *Oryza rufipogon Griff*. is involved in conferring high-temperature tolerance at the heading stage. Plant Biotechnol. J..

[30] Zhou H., Wang X., Huo C., Wang H., An Z., Sun D., Liu J., Tang W., Zhang B. (2019). A quantitative proteomics study of early heat-regulated proteins by two-dimensional difference gel electrophoresis identified *OsUBP21* as a negative regulator of heat stress responses in rice. Proteomics.

[31] Liu X., Lyu Y., Yang W., Yang Z., Lu S., Liu J. (2020). A membrane-associated NAC transcription factor *OsNTL3* is involved in thermotolerance in rice. Plant Biotechnol. J..

[32] Liao M., Ma Z., Kang Y., Zhang B., Gao X., Yu F., Yang P., Ke Y. (2023). ENHANCED DISEASE SUSCEPTIBILITY 1 promotes hydrogen peroxide scavenging to enhance rice thermotolerance. Plant Physiol..

[33] Liu G., Zha Z., Cai H., Qin D., Jia H., Liu C., Qiu D., Zhang Z., Wan Z., Yang Y. (2020). Dynamic transcriptome analysis of anther response to heat stress during anthesis in thermotolerant rice (*Oryza sativa* L.). Int. J. Mol. Sci..

[34] Vitoriano C.B., Calixto C.P.G. (2021). Reading between the Lines: RNA-seq data mining reveals the alternative message of the rice leaf transcriptome in response to heat stress. Plants.

[35] Mittler R., Zandalinas S.I., Fichman Y., Van Breusegem F. (2022). Reactive oxygen species signalling in plant stress responses. Nat. Rev. Mol. Cell Biol..

[36] Zhao Q., Zhou L., Liu J., Du X., Asad M., Huang F., Pan G., Cheng F. (2018). Relationship of ROS accumulation and superoxide dismutase isozymes in developing anther with floret fertility of rice under heat stress. Plant Physiol. Biochem..

[37] Volkov R.A., Panchuk I.I., Mullineaux P.M., Schöffl F. (2006). Heat stress-induced H_2_O_2_ is required for effective expression of heat shock genes in *Arabidopsis*. Plant Mol. Biol..

[38] Cvikrová M., Gemperlová L., Dobrá J., Martincová O., Prásil I.T., Gubis J., Vanková R. (2012). Effect of heat stress on polyamine metabolism in proline-over-producing tobacco plants. Plant Sci..

[39] Hanif S., Saleem M.F., Sarwar M., Irshad M., Shakoor A., Wahid M.A., Khan H.Z. (2021). Biochemically triggered heat and drought stress tolerance in rice by proline application. J. Plant Growth Regul..

[40] Sailaja B., Subrahmanyam D., Neelamraju S., Vishnukiran T., Rao Y.V., Vijayalakshmi P., Voleti S.R., Bhadana V.P., Mangrauthia S.K. (2015). Integrated physiological, biochemical, and molecular analysis identifies important traits and mechanisms associated with differential response of rice genotypes to elevated temperature. Front. Plant Sci..

[41] Cai H., Wang H., Zhou L., Li B., Zhang S., He Y., Guo Y., You A., Jiao C., Xu Y. (2023). Time-series transcriptomic analysis of contrasting rice materials under heat stress reveals a faster response in the tolerant cultivar. Int. J. Mol. Sci..

[42] Bokszczanin K.L., Fragkostefanakis S., Solanaceae Pollen Thermotolerance Initial Training Network (SPOT-ITN) Consortium (2013). Perspectives on deciphering mechanisms underlying plant heat stress response and thermotolerance. Front. Plant Sci..

[43] Mittler R., Finka A., Goloubinoff P. (2012). How do plants feel the heat?. Trends Biochem. Sci..

[44] Li Z., Ye X. (2022). Transcriptome response of maize (*Zea mays* L.) seedlings to heat stress. Protoplasma.

[45] Liu J.-X., Howell S.H. (2010). Endoplasmic reticulum protein quality control and its relationship to environmental stress responses in plants. Plant Cell.

[46] Chen Y., Deng C., Xu Q., Chen X., Jiang F., Zhang Y., Hu W., Zheng S., Su W., Jiang J. (2022). Integrated analysis of the metabolome, transcriptome and miRNome reveals crucial roles of auxin and heat shock proteins in the heat stress response of loquat fruit. Sci. Hortic..

[47] Kotak S., Larkindale J., Lee U., von Koskull-Döring P., Vierling E., Scharf K. (2007). Complexity of the heat stress response in plants. Curr. Opin. Plant Biol..

[48] Jacob P., Hirt H., Bendahmane A. (2017). The heat-shock protein/chaperone network and multiple stress resistance. Plant Biotechnol. J..

[49] Surabhi G.-K., Badajena B., Wani S.H. (2020). Chapter 10–Recent advances in plant heat stress transcription factors. Transcription Factors for Abiotic Stress Tolerance in Plants.

[50] Zhang H., Zhu J., Gong Z., Zhu J.K. (2022). Abiotic stress responses in plants. Nat. Rev. Genet..

[51] Licausi F., Ohme-Takagi M., Perata P. (2013). APETALA2/Ethylene Responsive Factor (AP2/ERF) transcription factors: Mediators of stress responses and developmental programs. New Phytol..

[52] Zhu Q., Zhang J., Gao X., Tong J., Xiao L., Li W., Zhang H. (2010). The *Arabidopsis* AP2/ERF transcription factor *RAP2.6* participates in ABA, salt and osmotic stress responses. Gene.

[53] Cui M., Zhang W., Zhang Q., Xu Z., Zhu Z., Duan F., Wu R. (2011). Induced over-expression of the transcription factor *OsDREB2A* improves drought tolerance in rice. Plant Physiol. Biochem..

[54] Oh S.J., Kim Y.S., Kwon C.W., Park H.K., Jeong J.S., Kim J.K. (2009). Overexpression of the transcription factor *AP37* in rice improves grain yield under drought conditions. Plant Physiol..

[55] Jin Y., Pan W., Zheng X., Cheng X., Liu M., Ma H., Ge X. (2018). *OsERF101*, an ERF family transcription factor, regulates drought stress response in reproductive tissues. Plant Mol. Biol..

[56] Chen J.Q., Meng X.P., Zhang Y., Xia M., Wang X.P. (2008). Over-expression of *OsDREB* genes lead to enhanced drought tolerance in rice. Biotechnol. Lett..

[57] Fukao T., Yeung E., Bailey-Serres J. (2011). The submergence tolerance regulator SUB1A mediates crosstalk between submergence and drought tolerance in rice. Plant Cell.

[58] Song Y., Ai C., Jing S., Yu D. (2010). Research progress on functional analysis of rice WRKY genes. Rice Sci..

[59] Upadhyaya G., Das A., Ray S. (2021). A rice R2R3-MYB (*OsC1*) transcriptional regulator improves oxidative stress tolerance by modulating anthocyanin biosynthesis. Physiol. Plant..

[60] Chen S., Cao H., Huang B., Zheng X., Liang K., Wang G.-L., Sun X. (2022). The WRKY10-VQ8 module safely and effectively regulates rice thermotolerance. Plant Cell Environ..

[61] Saad A.S.I., Li X., Li H., Huang T., Gao C., Guo M.-W., Cheng W., Zhao G., Liao Y. (2013). A rice stress-responsive NAC gene enhances tolerance of transgenic wheat to drought and salt stresses. Plant Sci..

[62] Yang W.T., Baek D., Yun D.-J., Hwang W.H., Park D.S., Nam M.H., Chung E.S., Chung Y.S., Yi Y.B., Kim D.H. (2014). Overexpression of *OsMYB4P*, an R2R3-type MYB transcriptional activator, increases phosphate acquisition in rice. Plant Physiol. Biochem..

[63] Redillas M.C.F.R., Jeong J.S., Kim Y.S., Jung H., Bang S.W., Choi Y.D., Ha S.-H., Reuzeau C., Kim J.-K. (2012). The overexpression of *OsNAC9* alters the root architecture of rice plants enhancing drought resistance and grain yield under field conditions. Plant Biotechnol. J..

[64] Wang Y., Liao Y., Quan C., Li Y., Yang S., Ma C., Mo Y., Zheng S., Wang W., Xu Z. (2022). C2H2-type zinc finger *OsZFP15* accelerates seed germination and confers salinity and drought tolerance of rice seedling through ABA catabolism. Environ. Exp. Bot..

[65] Huang J., Sun S., Xu D., Lan H., Sun H., Wang Z., Bao Y., Wang J., Tang H., Zhang H. (2012). A TFIIIA-type zinc finger protein confers multiple abiotic stress tolerances in transgenic rice (*Oryza sativa* L.). Plant Mol. Biol..

[66] Wang C., Zhang Q., Shou H.-X. (2009). Identification and expression analysis of *OsHsfs* in rice. J. Zhejiang Univ. Sci. B.

[67] Mittal D., Chakrabarti S., Sarkar A., Singh A., Grover A. (2009). Heat shock factor gene family in rice: Genomic organization and transcript expression profiling in response to high temperature, low temperature and oxidative stresses. Plant Physiol. Biochem..

[68] Thao N.P., Chen L., Nakashima A., Hara S.-I., Umemura K., Takahashi A., Shirasu K., Kawasaki T., Shimamoto K. (2007). RAR1 and HSP90 form a complex with Rac/Rop GTPase and function in innate-immune responses in rice. Plant Cell.

[69] Chen L., Hamada S., Fujiwara M., Zhu T., Thao N.P., Wong H.L., Krishna P., Ueda T., Kaku H., Shibuya N. (2010). The Hop/Sti1-Hsp90 chaperone complex facilitates the maturation and transport of a PAMP receptor in rice innate immunity. Cell Host Microbe.

[70] Moon J.-C., Ham D.J., Hwang S.-G., Park Y.C., Lee C., Jang C.S. (2014). Molecular characterization of a heat inducible rice gene, *OsHSP1*, and implications for rice thermotolerance. Genes Genom..

[71] Chang Y., Nguyen B.H., Xie Y., Xiao B., Tang N., Zhu W., Mou T., Xiong L. (2017). Co-overexpression of the constitutively active form of *OsbZIP46* and ABA-activated protein kinase SAPK6 improves drought and temperature stress resistance in rice. Front. Plant Sci..

[72] Xiang J., Ran J., Zou J., Zhou X., Liu A., Zhang X., Peng Y., Tang N., Luo G., Chen X. (2013). Heat shock factor *OsHsfB2b* negatively regulates drought and salt tolerance in rice. Plant Cell Rep..

[73] Hoang T.V., Vo K.T.X., Rahman M.M., Choi S.H., Jeon J.S. (2019). Heat stress transcription factor *OsSPL7* plays a critical role in reactive oxygen species balance and stress responses in rice. Plant Sci..

[74] Kawahara Y., de la Bastide M., Hamilton J.P., Kanamori H., McCombie W.R., Ouyang S., Schwartz D.C., Tanaka T., Wu J., Zhou S. (2013). Improvement of the *Oryza sativa* Nipponbare reference genome using next generation sequence and optical map data. Rice.

[75] Love M.I., Huber W., Anders S. (2014). Moderated estimation of fold change and dispersion for RNA-seq data with DESeq2. Genome Biol..

