# Supplementary material for: Transcriptome Analysis Reveals the Dynamic and Rapid Transcriptional Reprogramming Involved in Heat Stress and Identification of Heat Response Genes in Rice
Source: Int J Mol Sci. 2023 Sep 30;24(19):14802. doi: 10.3390/ijms241914802 (PMC10572967; doi:10.3390/ijms241914802)

Table S1. Summary of the quality control of sequencing data.

Sample ID	Clean Reads	Mapped Reads (Ratio)	Unique Mapped Reads (Ratio)	GC Content	Clean reads Q30	Reads mapped to exon
T2_0_1	47,260,196	44,490,905 (94.14%)	43,160,390 (91.33%)	56.01%	94.46%	91.20%
T2_0_2	43,649,364	41,118,257 (94.20%)	40,175,621 (92.04%)	55.22%	94.61%	92.18%
T2_0_3	43,849,292	41,331,569 (94.26%)	40,422,417 (92.18%)	55.69%	93.95%	92.21%
T2_0.5_1	49,073,396	46,395,176 (94.54%)	44,731,092 (91.15%)	56.06%	94.20%	87.62%
T2_0.5_2	46,253,258	43,477,939 (94.00%)	41,758,862 (90.28%)	55.89%	93.37%	86.41%
T2_0.5_3	47,329,488	44,572,720 (94.18%)	42,969,880 (90.79%)	55.89%	94.20%	86.15%
T2_1_1	56,216,804	52,785,274 (93.90%)	50,959,608 (90.65%)	55.67%	94.26%	83.70%
T2_1_2	47,158,872	44,176,128 (93.68%)	43,123,564 (91.44%)	56.82%	93.51%	88.18%
T2_1_3	50,164,248	47,189,091 (94.07%)	44,777,254 (89.26%)	56.45%	94.10%	85.97%
T2_3_1	41,856,706	38,641,320 (92.32%)	36,542,315 (87.30%)	55.10%	93.67%	85.61%
T2_3_2	51,186,706	47,381,427 (92.57%)	46,246,415 (90.35%)	54.48%	94.54%	86.72%
T2_3_3	47,850,052	44,616,916 (93.24%)	43,569,883 (91.06%)	54.83%	94.73%	86.84%
T2_8_1	53,401,364	49,719,671 (93.11%)	47,427,364 (88.81%)	54.31%	94.52%	88.29%
T2_8_2	59,082,624	54,581,259 (92.38%)	53,225,770 (90.09%)	53.99%	94.49%	86.51%
T2_8_3	46,562,980	42,738,318 (91.79%)	40,286,179 (86.52%)	54.30%	94.11%	85.85%
T2_12_1	39,566,208	36,254,804 (91.63%)	35,289,554 (89.19%)	53.52%	94.21%	88.93%
T2_12_2	45,393,134	41,718,290 (91.90%)	40,650,173 (89.55%)	53.45%	94.59%	87.86%
T2_12_3	46,527,202	42,715,728 (91.81%)	41,649,872 (89.52%)	53.49%	94.48%	85.52%
T21_0_1	47,622,466	45,168,335 (94.85%)	44,128,550 (92.66%)	56.10%	94.41%	93.29%
T21_0_2	40,934,920	38,714,980 (94.58%)	37,878,452 (92.53%)	56.29%	94.68%	92.68%
T21_0_3	49,423,142	46,825,465 (94.74%)	45,740,395 (92.55%)	55.87%	94.52%	92.24%
T21_0.5_1	42,472,770	40,288,333 (94.86%)	38,398,196 (90.41%)	56.29%	94.40%	88.83%
T21_0.5_2	53,294,506	50,292,699 (94.37%)	48,906,653 (91.77%)	56.95%	93.93%	91.46%
T21_0.5_3	40,282,720	38,019,750 (94.38%)	36,956,030 (91.74%)	56.57%	94.06%	90.85%
T21_1_1	45,809,032	43,327,911 (94.58%)	42,179,783 (92.08%)	55.94%	93.96%	90.46%
T21_1_2	59,514,344	56,315,248 (94.62%)	53,698,847 (90.23%)	56.00%	94.34%	89.56%
T21_1_3	42,997,082	40,661,770 (94.57%)	38,781,332 (90.20%)	55.64%	94.24%	89.42%
T21_3_1	49,458,296	46,301,067 (93.62%)	44,535,205 (90.05%)	55.07%	94.07%	88.71%
T21_3_2	44,106,466	41,426,210 (93.92%)	39,820,701 (90.28%)	54.77%	94.12%	90.05%
T21_3_3	45,985,288	43,204,961 (93.95%)	41,200,211 (89.59%)	55.47%	94.64%	89.70%
T21_8_1	47,197,744	42,855,595 (90.80%)	40,744,886 (86.33%)	53.57%	94.34%	83.20%
T21_8_2	39,038,336	35,191,366 (90.15%)	33,074,368 (84.72%)	53.05%	94.44%	80.17%
T21_8_3	39,019,810	35,762,546 (91.65%)	34,213,820 (87.68%)	53.60%	94.61%	86.21%
T21_12_1	41,976,638	38,457,844 (91.62%)	37,519,382 (89.38%)	53.09%	93.61%	89.38%
T21_12_2	56,681,874	52,364,361 (92.38%)	49,518,075 (87.36%)	52.97%	94.24%	86.67%
T21_12_3	46,316,740	42,716,324 (92.23%)	41,677,632 (89.98%)	52.93%	94.44%	88.57%

Table S2: Primers used in the current study.

Primer names	Forward (5'-3')	Reverse (5'-3')
OsActin	CCGAGCGGGAAATTGTGAG	CTATGAAGGAAGGCTGGAAGAGG
Os05g23740	CCAGGCGATGTGAAGGGTAA	CAGCATCGATGACATCACCT
Os01g09550	GCACGTCTTCGAGCACAAAA	TGGCTGGTAAGGCCATTCTG
Os03g50860	GTGGAGTGTGTGGAGAGTGG	TGTCGTCTGCCTGCTCATTT
Os07g37180	GGACCCCGGTTTCCATTGAT	GTATCCCGCGCATTTGTGAC
Os02g02880	CAATCTGGTGGTGTAAGACC	TCCAGGAGAAGTATGACC
Os03g60080	CATGGTCCCGTTCTGAGGTG	CACACGTTGCAGCATCGATC
Os09g35790	GTCCAGCTCCAGCCAAACGAT	CCTTACGCCACTACCGCATTCC
Os07g04700	GCTGCTGTCAACACCTTTGG	CTCTGTGTGCGACCAGGAAT
Os08g38086	GAGGCGAAAGAGGGTGAACA	CCACCTCACCCTCTGTTGTG
Os03g32160	TGATCCCTCGAGTGCATTGG	GGTGACTGGTGCAATCCTCA

Figure S1: Validation of RNA-seq data via RT-qPCR.

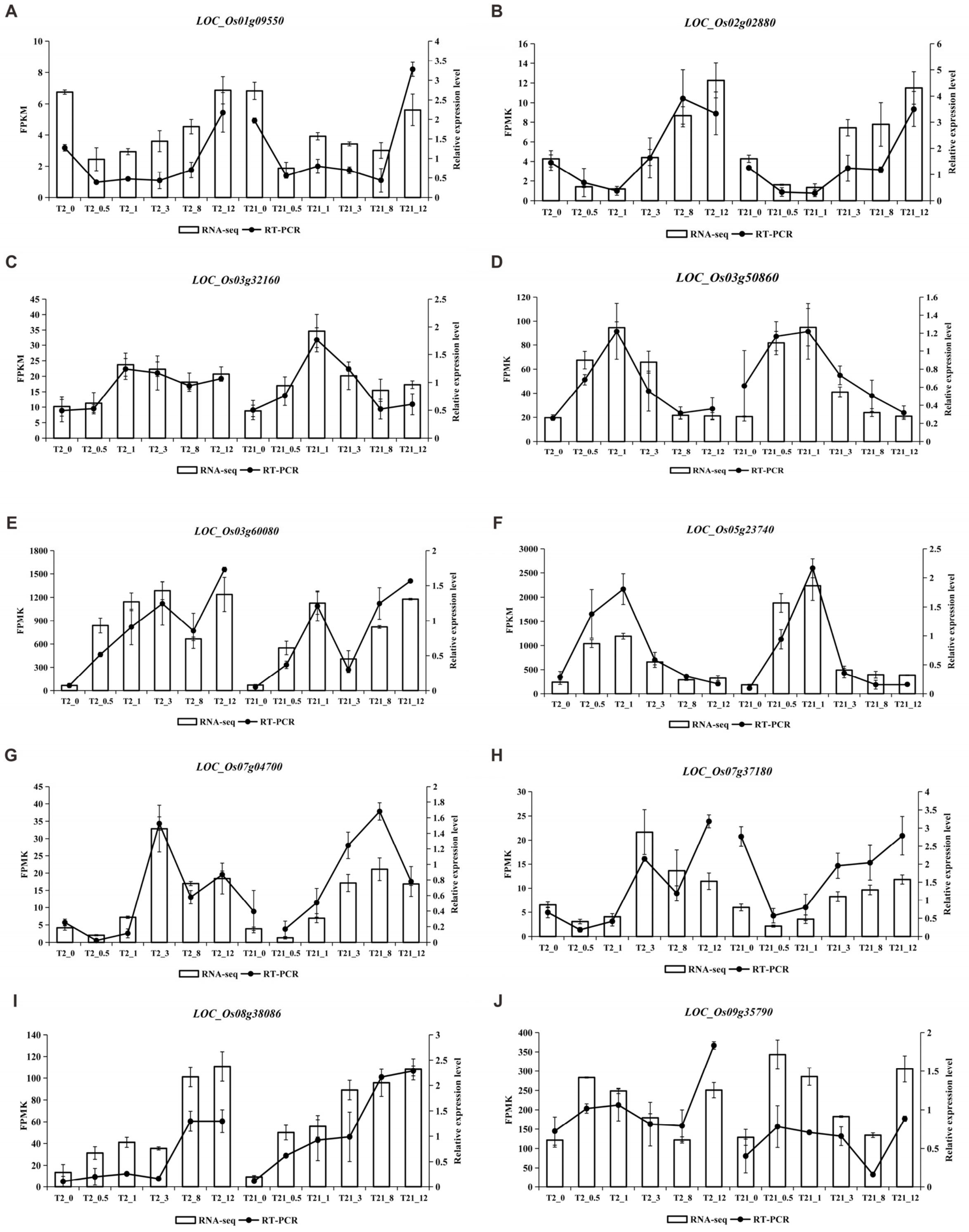

Figure S2: The volcano plot depicts all statistically significant DEGs identified by T21 vs. T2 before heat treatment.

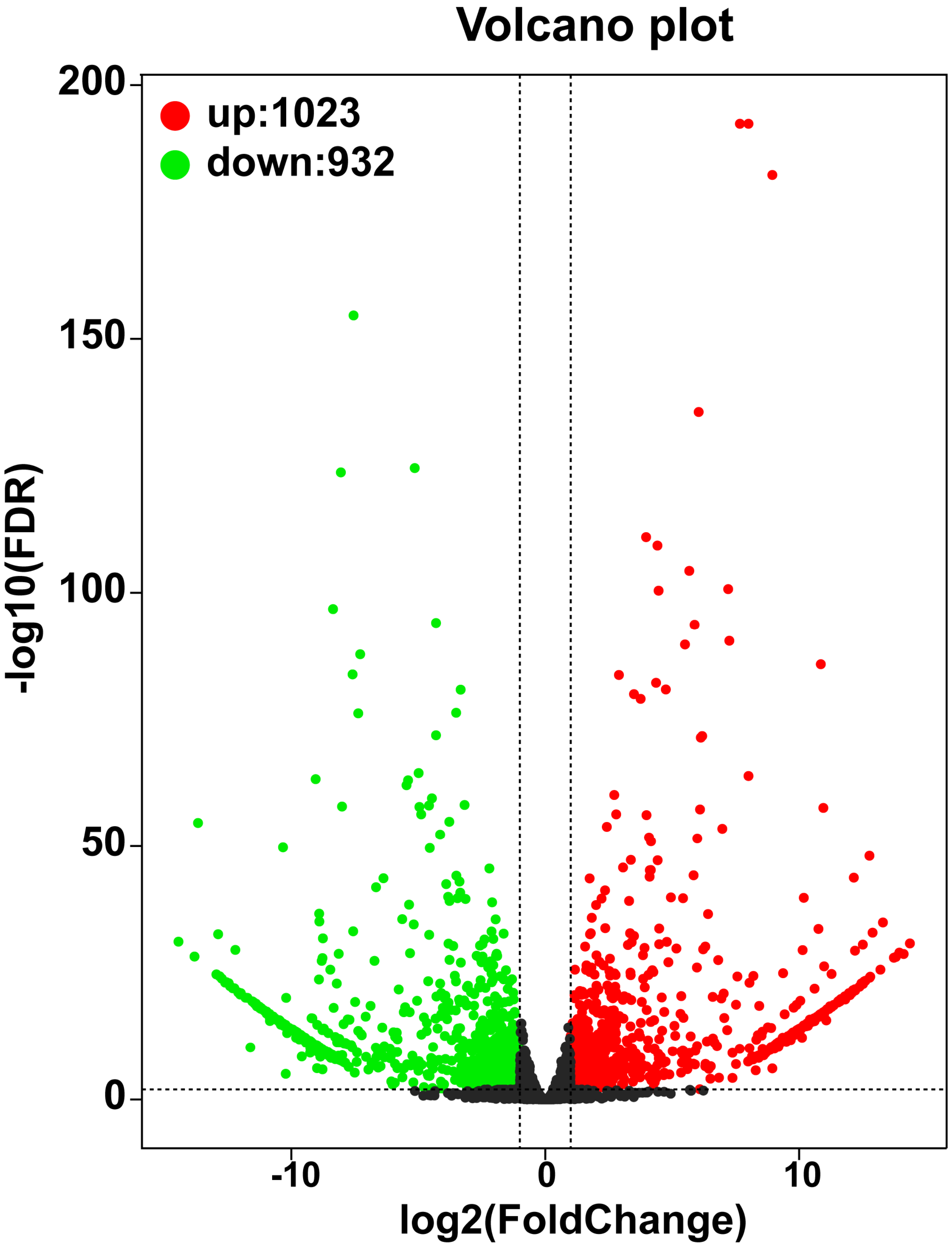

Supplement: Supplementary file 1 [file ijms-24-14802-s001.zip › ijms-2634923-supplementary.pdf]
